# Pyoderma Gangrenosum in a 75-Year-Old Male With Follicular Diffuse Large B-cell Lymphoma

**DOI:** 10.7759/cureus.67234

**Published:** 2024-08-19

**Authors:** Andrew P Bell, Michelle K Custer, Steven Presley

**Affiliations:** 1 Medicine, Edward Via College of Osteopathic Medicine, Auburn, USA; 2 Simulation Center, Edward Via College of Osteopathic Medicine, Auburn, USA; 3 Internal Medicine, East Alabama Medical Center, Opelika, USA

**Keywords:** immunosupression, neutrophilic dermatosis, ulcerative lesions, wound vacuum, dermal necrosis, large b cell lymphomas, pyoderma gangenosum

## Abstract

Pyoderma gangrenosum (PG) is a rare neutrophilic dermatosis that characteristically presents with progressive ulcerative lesions. The association of PG with hematological malignancies remains unclear due to its varied clinical presentation. Herein, we report the unusual case of PG in a 75-year-old male with stage III follicular diffuse large B-cell lymphoma. Seven days subsequent to his first dose of rituximab, cyclophosphamide, doxorubicin, vincristine, prednisolone (R-CHOP) therapy, he presented to the emergency department with generalized malaise, bilateral lower extremity edema, and ecchymoses with ulcerative wounds on the dorsal of his feet. Due to the rapid progression of the patient’s dermatological manifestations and declining clinical status, he required serial surgical wound debridement and a biopsy, which revealed an occlusive vasculopathy with dermal and epidermal necrosis. These pathological findings, along with the patient’s clinical presentation, led to the diagnosis of PG. The patient was treated with negative pressure wound therapy, steroids, and tacrolimus ointment, which led to a marked improvement in the appearance of the patient’s dermatological features and clinical status.

## Introduction

Follicular diffuse large B-cell lymphoma is a non-Hodgkin lymphoma that expresses follicular and diffuse large B-cell histological patterns [1]. Painless peripheral lymphadenopathy is a notable presentation of this malignancy [1]. However, cutaneous manifestations, such as vesicular or ulcerative rashes, pruritis, and malaise, are unusual manifestations of lymphoma [1].

Pyoderma gangrenosum (PG) is an uncommon inflammatory and ulcerative dermatological condition that is often correlated with other immunological disorders such as rheumatoid arthritis and inflammatory bowel disease [2]. Although there have been reported cases of PG in association with hematological malignancies such as myelodysplastic syndrome, monoclonal gammopathy of undetermined significance, acute leukemia, chronic leukemia, and non-Hodgkin lymphoma, the relationship between them is not well understood [3].

PG characteristically presents as a rapidly progressive ulcerative lesion with undermined violaceous or erythematous borders surrounding a necrotic base and purulent exudate [2]. PG typically presents in individuals around 45 years of age, and it is estimated to affect less than 10 persons per million per year globally [4]. Although the etiology of PG remains unknown, it is believed to derive from an autoimmune process [2]. PG is classified as a neutrophilic dermatosis, which contributes to the development of ulcerative cutaneous lesions [2]. Here, we present a case of stage III follicular diffuse large B-cell lymphoma treated with rituximab, cyclophosphamide, doxorubicin, vincristine, prednisolone (R-CHOP) therapy associated with a clinical scenario suspicious of PG.

## Case presentation

A 75-year-old man with stage III follicular diffuse large B-cell lymphoma presented to the emergency department due to dyspnea. Upon examination, the patient was noted to have a right pleural effusion, attributed to his lymphoma, and subsequently underwent thoracentesis, which revealed bloody fluid. After receiving appropriate treatments and following improvement in his clinical status, he received his first dose of R-CHOP therapy for his lymphoma prior to discharge. Following two weeks, the patient presented back to the emergency department due to generalized fatigue and pain in his feet.

Evaluation of the patient revealed altered mental status, a blood pressure of 83/44 mmHg, and a respiratory rate of 28 breaths per minute. He was admitted to the intensive care unit for the management of septic shock in the setting of chemotherapy-induced neutropenia. Bilateral lower extremity edema was noted during the physical exam. Additionally, his left foot displayed localized swelling and ecchymoses with a 10-cm ulcerative lesion located dorsally. Purulence was noted over the second, third, and fourth anterior metatarsal heads up to the ankle. Similarly, the right foot demonstrated an 8-cm erythematous ulcerative lesion that extended across the second, third, and fourth anterior metatarsal heads.

The bilateral foot wounds were debrided, and a biopsy with culture of the lesions was performed, which revealed *Streptococcus agalactiae*. The patient’s blood culture revealed *Streptococcus agalactiae* as well. He began antibiotic therapy with piperacillin-tazobactam. The patient’s wounds were cleaned with sterile saline, followed by moist, wet-to-dry dressing changes daily.

Despite wound care and completion of the antibiotic regimen, the patient’s dermatological manifestations continued to progress and showed no improvement in clinical presentation. A second debridement was performed, which extended to the level of the tendons. At this time, wound culture was unrevealing. However, excisional biopsy displayed occlusive vasculopathy with dermal and epidermal necrosis. The patient’s clinical presentation, followed by the biopsy results, led to a clinical suspicion of PG.

Intralesional triamcinolone injections (6 mg/mL) were initiated. Routine dressing changes and wound care were performed for one week before the patient transitioned to bilateral negative pressure wound therapy. Triamcinolone injections followed by tacrolimus ointment were continued upon the patient’s discharge to a skilled nursing facility for further care and rehabilitation.

At the skilled nursing facility, he completed 20 mg of prednisone daily for five days and was started on mycophenolate mofetil 500 mg twice daily. Two weeks following treatment, the bilateral foot wounds began to show improvement but remained open, requiring continued use of negative pressure wound therapy changed three times weekly at discharge.

**Figure 1:**
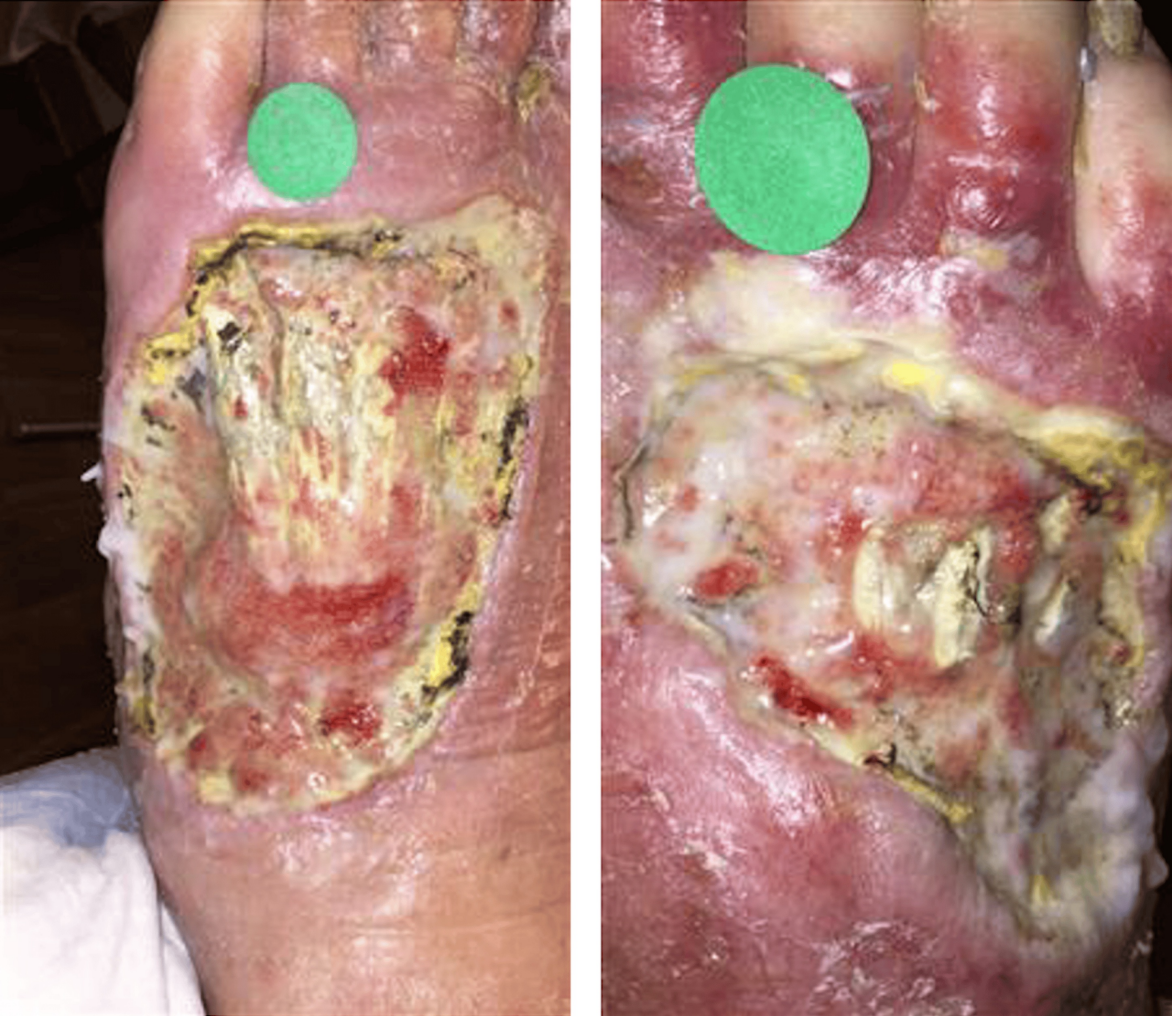
Bilateral foot wounds at six-week outpatient follow-up (A) Dorsum of the left foot showing localized swelling and an ulcerative lesion with an indurated border and purulent exudate on an erythematous base. Granulation tissue is diffusely present. (B) Dorsum of the right foot showing localized swelling and an ulcerative lesion with an indurated border and purulent exudate on an erythematous base. Granulation tissue is diffusely present.

**Figure 2:**
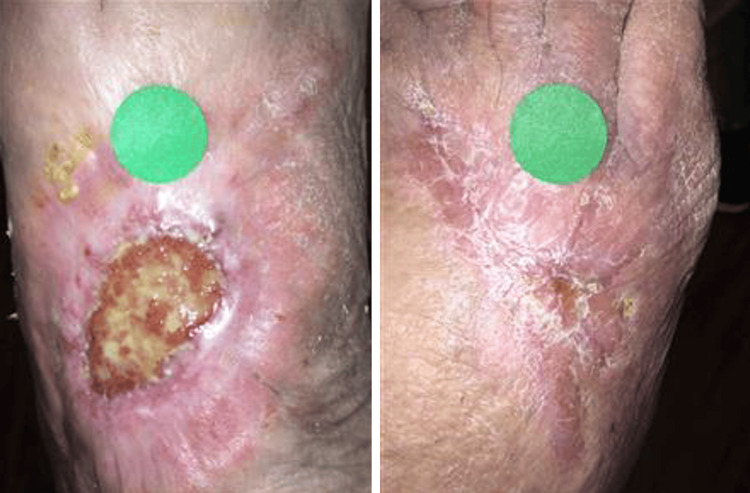
Bilateral foot wounds at five-month outpatient follow-up (A) Dorsum of the left foot showing a reduction in the size of the ulcerative lesion, epithelialization on the wound surface, decreased purulent exudate, and reduced swelling in the surrounding tissues. (B) Dorsum of the right foot showing a reduction in the size of the ulcerative lesion, epithelialization on the wound surface, decreased erythema, minimized purulent exudate, and reduced swelling.

As displayed in Figure 1A and Figure 1B, the patient’s bilateral foot wounds showed marked improvement at his six-week outpatient follow-up. The wound vacuum was discontinued, and daily dressing changes were continued. At the patient's five-month outpatient follow-up, continued improvement was noted in the patient’s bilateral foot wounds, which is shown in Figure 2. The patient was instructed to maintain treatment and routine dressing changes.

## Discussion

The pathogenesis of PG is believed to be due to dysregulation of the innate and adaptive immune systems. While the true connection between individuals with hematological malignancies and PG requires further studies, this immune dysregulation and the inflammatory cytokine profile of tumor necrosis factor-alpha (TNF-α), IL-8, and vascular endothelial growth factor (VEGF) are suspected to be linked [5].

It is believed that TNF-α plays a key role in stimulating IL-8, a neutrophil attractant [5]. Likewise, increased levels of TNF-a, IL-8, and VEGF have been demonstrated in hematological diseases [5]. The co-expression of PG and follicular diffuse large B-cell lymphoma may be ascribed to a shared cytokine profile such as TNF-a, IL-8, and VEGF [5]. It is also possible that treatment for hematological malignancies, such as R-CHOP therapy, could exacerbate an autoimmune response, leading to the development of PG.

Georgakopoulos et al. report a case of PG that developed in a patient who initially showed no signs of PG after completing six doses of R-CHOP therapy for the treatment of diffuse large B-cell lymphoma [6]. However, two years later, the patient developed PG after being treated with rituximab alone for the treatment of rheumatoid arthritis [6]. This case may suggest an association between the use of R-CHOP therapy and the development of PG. Additionally, it highlights how PG can differ in its onset of cutaneous manifestation.

PG is primarily a diagnosis of exclusion with no true confirmatory test [7]. Clinical diagnosis requires the major criterion of a biopsy edge showing neutrophilic infiltrate along with at least four of the following minor criteria: exclusion of infection, pathergy, personal history of inflammatory bowel disease or inflammatory arthritis, history of papule, pustule, or vesicle that rapidly ulcerated, peripheral erythema, undermining border, and tenderness of ulcer, multiple ulcerations with at least one occurring on an anterior lower leg, cribriform or wrinkled paper scars at sites of healed ulcers, and a decrease in ulcer size within one month of immunosuppressive therapy [8-12].

Treatment guidelines recommend immune suppressive therapy with topical or intralesional high-potency steroids, along with routine cleaning and dressing changes initially. If unresponsive to initial therapy, switching to topical tacrolimus or oral dapsone is recommended [7].

## Conclusions

Although PG has been associated with other autoimmune conditions, such as rheumatoid arthritis and inflammatory bowel disease, its relationship with hematological disease processes lacks complete understanding, emphasizing the need for further research. Cutaneous manifestation of follicular diffuse large B-cell lymphoma in the form of a solid lump under the skin is an uncommon finding necessitating prompt diagnostic tests and treatment modalities. Possible causation of PG due to rituximab use in R-CHOP therapy must be considered but lacks confirmatory testing. Multidisciplinary collaboration is critical in providing an accurate diagnosis and guiding proper therapeutic interventions for optimal patient care.

## References

[1] Sapkota S, Shaikh H (2023). Non-Hodgkin Lymphoma. http://www.ncbi.nlm.nih.gov/books/NBK559328/.

[2] Maverakis E, Marzano AV, Le ST (2020). Pyoderma gangrenosum. Nat Rev Dis Primers.

[3] Montagnon CM, Fracica EA, Patel AA (2020). Pyoderma gangrenosum in hematologic malignancies: a systematic review. J Am Acad Dermatol.

[4] Su WP, Davis MD, Weenig RH, Powell FC, Perry HO (2004). Pyoderma gangrenosum: clinicopathologic correlation and proposed diagnostic criteria. Int J Dermatol.

[5] Padhi T, Pradhan S, Pradhan K, Kumar SK (2016). Pyoderma gangrenosum associated with mantle cell lymphoma. Indian Dermatol Online J.

[6] Georgakopoulos JR, Rohekar G, Lovegrove FE (2024). A case of rituximab-induced pyoderma gangrenosum. JAAD Case Rep.

[7] Schadt C (2022). Pyoderma gangrenosum: pathogenesis, clinical features, and diagnosis. UpToDate.

[8] Haase LR, Nelson GB, Klyce WB, Cavaliere CM, Bafus BT (2022). Pyoderma gangrenosum affecting the dorsal hand: a case report. Plast Reconstr Surg Glob Open.

[9] David F, Lopes Freitas R, Brás-Cruz R, Rocha J, Rosário C (2022). A case of recurrent idiopathic pyoderma gangrenosum. Cureus.

[10] Mohtadi M, Alocha H, Mahmoud A, Perez C, Lovaas C (2024). From unassuming to unbelievable: a case report of pyoderma gangrenosum. Cureus.

[11] Aziret M, Kara Ş, Yaldız M, Köse N, Aşıkuzunoğlu F, Cevrioğlu AS (2021). An extensive pyoderma gangrenosum mimicking necrotizing fasciitis: an unusual case report. Int J Surg Case Rep.

[12] Ashchyan HJ, Butler DC, Nelson CA (2018). The association of age with clinical presentation and comorbidities of pyoderma gangrenosum. JAMA Dermatol.

